# The Prevalence of Dyslexia in Primary School Children and Their Chinese Literacy Assessment in Shantou, China

**DOI:** 10.3390/ijerph17197140

**Published:** 2020-09-29

**Authors:** Yuhang Lin, Xuanzhi Zhang, Qingjun Huang, Laiwen Lv, Anyan Huang, Ai Li, Kusheng Wu, Yanhong Huang

**Affiliations:** 1 Mental Health Center, Shantou University Medical College, Shantou 515065, China; 18yxlin@stu.edu.cn (Y.L.); zhangxuanzhi2018@163.com (X.Z.); huangqj@stu.edu.cn (Q.H.); 19ayhuang@stu.edu.cn (A.H.); ailicz@163.com (A.L.); 2 Master of Public Health Education Center, Shantou University Medical College, Shantou 515041, China; laiwen@stu.edu.cn; 3 Department of Preventive Medicine, Shantou University Medical College, Shantou 515041, China

**Keywords:** Chinese children, developmental dyslexia, prevalence, Chinese literacy

## Abstract

The epidemiological studies of Chinese developmental dyslexia (DD) in China are still limited. In addition, literacy assessment has seldom been performed for children with dyslexia, due to lack of uniform assessment tools. This study was aimed at investigating the prevalence rate of children with dyslexia, and to evaluate their Chinese reading ability. A total of 2955 students aged 7–12 years were enrolled by randomized cluster sampling. The study was divided into three stages. In stage I, all participating students were asked to finish the Combined Raven Test (CRT) and Chinese Vocabulary Test and Assessment Scale. In stage II, the Chinese teachers and parents of the children with suspected dyslexia were interviewed by psychiatrists, and finished the Dyslexia Checklist for Chinese Children (DCCC). In stage III, these children were evaluated by child psychiatrists for the diagnosis with or without dyslexia, according to the fifth edition of Diagnostic and Statistical Manual of Mental Disorders (DSM-5), and their Chinese literacy was further evaluated by using the Chinese Reading Ability Test (CRAT). The prevalence rate of children with dyslexia was 5.4% in Shantou city, 8.4% in boys and 2.3% in girls, with a gender ratio of 3.7:1.0. Children with dyslexia scored lower in all the five subscales of the CRAT tests. including phonological awareness, morphological awareness, rapid automatized naming, orthographic awareness, and reading ability than the control group (all *p* < 0.001). This study suggested that the prevalence rate of Chinese dyslexia in Shantou city is roughly equivalent to that previously reported in China. Children with dyslexia have a relatively lower Chinese reading ability in all assessments.

## 1. Introduction

Developmental dyslexia (DD) is a neurodevelopmental disorder, characterized by reading difficulties that include inaccurate or slow and effortful word reading, poor decoding, and poor spelling abilities [1]. According to the fifth edition of Diagnostic and Statistical Manual of Mental Disorders (DSM-5), dyslexia is classified as a special learning disorder, accounting for about 80% of special learning disabilities [2]. Even though children with DD have no difference in education and sociocultural resources compared to typically developed children, their reading abilities are below the levels expected for their ages. Children with dyslexia may have other mental problems and academic setbacks, which may affect their future work and life [3,4,5]. An Italian study showed that university students with dyslexia experience higher levels of somatic complaints, social problems, lower self-esteem, and higher depression scores than typically developed students matched for gender, education, and academic discipline [6]. In addition, a 30-year, population-based longitudinal study in the United States indicated that children with dyslexia at age 7 were less likely to attain a higher level of education and income when they grow up compared to normal children with average or above reading achievement [7]. Early identification of dyslexia can lead to earlier intervention to improve academic achievement and life skills. Therefore, dyslexia is an important public health problem worthy of attention, and early screening and diagnosis for dyslexia are much crucial.

DD is widely prevalent, occurring for all languages in the world [8]. Studies on dyslexia in English-speaking countries have a history of more than a century [9]. In contrast, there have been few studies in non-English-speaking countries [10]. The incidence of dyslexia in the English-speaking world is reported to be around 5% to 10% in clinic patients and 17.5% in school students [11,12]. Chinese is one of the most populous languages in the world, but the research of Chinese dyslexia lags behind that of English. In the 1970s, DD was thought to occur only in languages where phonetic characters were used, such as English, and was extremely rare among speakers of Asian languages such as Chinese and Japanese [13]. Later on, reports from Singapore, Taiwan, and mainland China confirmed the existence of Chinese developmental dyslexia, and found that its incidence was not low [14].

The prevalence of dyslexia varies by languages and countries. Compared with alphabetic languages, in which letters represent phonemes, the smallest written units in Chinese are characters representing monosyllabic morphemes. According to surveys, the prevalence rate of Chinese language dyslexia in school-age children is from 3.0% to 12.6% [15]. Apart from this, due to different methods, tests, and definitions adopted for diagnosis, there existed differences in the incidence of Chinese dyslexia reported in the literature [16]. Epidemiological surveys have reported that the prevalence rates of DD in Guangzhou (south China), Taipei (southeast China), Xinjiang (northwest China in Uyghur), and Qianjiang (middle China) are 5.4%, 9.7%, 7.0%, and 3.9%, respectively [14,17,18,19]. However, these studies adopted inconsistent diagnostic criteria and confirmation tests for dyslexia to estimate the prevalence rate. The majority of the literature reported in China has usually used parents-based scales to confirm dyslexia, accompanying by the involvement of child psychiatrists in the diagnosis, which did not measure the students’ actual reading level. In comparison to English, the questionnaires and tests that are available for the screening and diagnosis of dyslexia to assess Chinese-speaking individuals are much more limited. Therefore, this study will adopt a newly developed assessment tool combined with the typical diagnosis according to DSM-5, by child psychiatrists, to assess the prevalence of dyslexia in primary school children in Shantou city, China, and their real reading ability.

## 2. Materials and Methods

### 2.1. Study Design and Participants

This study was conducted in Shantou city located in the southeast of China, which has a population of 5,579,204 with area of 2199 square kilometers. A cross-sectional epidemiological study was designed for the dyslexia screening, and followed by a case-control study for the reading ability comparison. Primary school students from second to fifth grades in the 3 primary schools based on a randomized cluster sampling method were all recruited from 2016 to 2017. We selected 855, 557 and 1289 students from the three schools respectively. The sex ratio for each grade is roughly 1:1 in the three schools. All subjects gave their informed consent for inclusion before they participated in the study. The study was conducted in accordance with the Declaration of Helsinki, and the protocol was approved by the Ethics Committee of Mental Health Center, Shantou University Medical College (No.: MSUMC-2016-023).

### 2.2. Screening and Diagnosis Procedure

**Figure 1:**
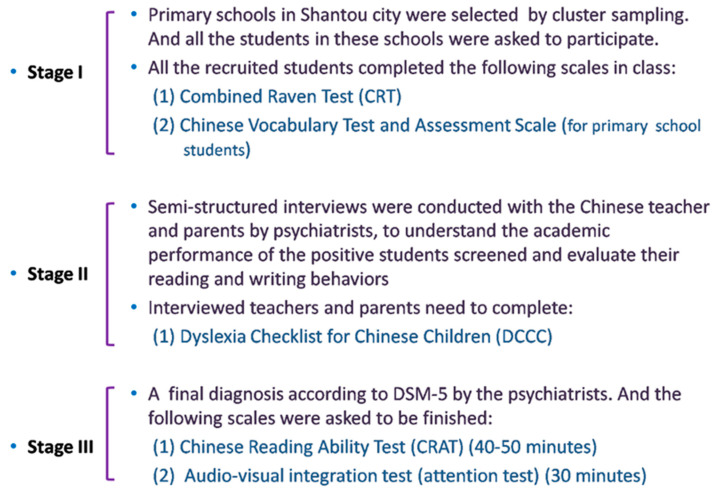
Flowchart of the three stages screening of diagnosis for children with dyslexia.

We applied the three-stage assessment of screening, interview, and diagnosis to reach a final diagnosis of dyslexia (Figure 1). The sample size of the screening was calculated as follows: $n=\frac{{u_{\alpha }}^{2}pq}{d^{2}}$. Where *u* is the statistic of the normal distribution, *α* is the value of significance; *p* is the prevalence of dyslexia in primary school students, and its value is 8% according to literature report; *q* =1 − *p*; *d* is the sensitivity level (margin error), and *d* = 0.15*p*. Then, 2047 was calculated based on the formula. Finally, we regarded 2252 as the minimum sample size after increasing it by 10%.

The first stage of screening was carried out at schools by two specifically trained investigators and five postgraduate students who are experienced in epidemiological survey. The process lasted nearly two weeks. All the students (*n* = 2955) from second to fifth grades in the selected three primary schools were asked to finish the Combined Raven Test (CRT) and Chinese Vocabulary Test and Assessment Scale (CVTAS) for primary school children. CRT (Cronbach’s α = 0.91) has been widely used in the screening children with dyslexia, in order to exclude children with intellectual problems [19,20,21]. CVTAS (Cronbach’s α = 0.75) is a widely used reading level test for screening Mandarin-speaking Chinese children for dyslexia [13]. Some students were excluded because of missing data for the main measurements (*n* = 240) or having a Raven’s intelligence quotient (IQ) below 80 (*n* = 14). Finally, 2701 cases were included in the final analysis. The academic achievements of students’ Chinese language were provided by Chinese teachers and checked carefully. The participants would be suggested as primary screening candidates for dyslexia if their Chinese language test was below the 25th percentile among all children in the same grade, or if their score on the CVTAS was lower more than one standard deviation.

The second stage of assessment was aiming at identifying children suspected to have dyslexia further, and this lasted three weeks approximately. In order to assess academic performance and reading level, the child psychiatrists interviewed the Chinese teacher and parents of the children with positive primary screenings using the Dyslexia Checklist for Chinese Children (DCCC). The DCCC, having a good reliability (Cronbach’s α = 0.974) and validity, is a practical rating scale for Chinese dyslexia identification [22]. The subject, identified as a suspected dyslexic child, should meet the following conditions: the score of Chinese language result was below the tenth percentile in the same grade, and there were at least two standard deviations above the mean score in the DCCC.

The third stage of diagnosis was carried out in the Mental Health Centre of Shantou University Medical College by specifically trained Child psychiatrists, and it lasted about two weeks. The child psychiatrists offered consulting services to each child with suspected dyslexia to exclude other mental disorders. According to the diagnostic criteria of the DSM-5, two child psychiatrists would make the diagnosis for all children with suspected dyslexia, also referring to the screening and interviewing results. If they had different opinions, a third psychiatrist would be involved in discussing and making the final diagnosis. The diagnosis was also furtherly confirmed by reading ability test, which is introduced in detail below.

### 2.3. The Diagnostic Criteria of Developmental Dyslexia

The screening process for children with developmental dyslexia is complex, with multiple criteria. The international classic model is the differential diagnosis model based on IQ—that is, there is no significant neurological and sensory organ damage in development. Therefore, the children’s intelligence is normal, and reading levels significantly lag behind the corresponding intelligence levels or ages at the same level of education. This diagnostic model mainly includes an intelligence test, questionnaire survey and reading level test. The diagnosis of children with dyslexia should be accompanied by a thorough clinical assessment and neuropsychological testing to rule out other possible explanations. According to the diagnostic criteria of the DSM-5, we diagnosed the children with dyslexia according to the following criteria: (1) IQ above 80 by the Raven IQ test; (2) at least 1 SD below the average level of their actual grade on a Chinese vocabulary test (reading level test); (3) according to head-teachers’ reports, Chinese language test had been below the tenth percentile among all children in the same grade for 6 months; (4) child psychiatrists found no suspected brain damage, uncorrected sensory impairment, or other mental or neurological disorders.

### 2.4. Chinese Reading Ability Test between Dyslexia and Normal Children

As further confirmation and assist, we conducted a literacy test, called the Chinese Reading Ability Test (CRAT), for the preliminarily diagnosed dyslexia children, with normally developing children from the same classes as controls. The CRAT includes five subscales on Chinese cognitive-linguistic measures of phonological awareness, morphological awareness, rapid automatized naming, orthographic awareness, and reading comprehension [23], which took about 45 min to complete for each student. There are three tests in the phonological awareness subscale: rhyme identification, onset identification, and tone identification, which are classical tests in the study of dyslexia in Chinese. Orthographic knowledge refers to the ability to understand writing habits in a particular language and to distinguish whether writing is correct or not. Reading comprehension tests included reading time, as well as oral and written questions. The scores of the five subscales of CRAT were calculated to identify whether there were differences of reading ability between dyslexia children and normally developing children.

### 2.5. Statistical Analysis

Two professionals used Epidata 3.1 (The EpiData Association, Odense, Denmark) independently to establish a database. Descriptive analyses of age, sex, and grade were performed using mean ± standard deviation (SD) and frequencies. Univariate analyses of dyslexic and non-dyslexic children were examined by *t*-test or *χ*^2^ test. Multivariate analysis was performed using logistic regression models to explore the relationship between five cognitive–linguistic skills and dyslexia. For all *p*-values, the significance level of double tails was 0.05, and all statistical analyses were implemented in SPSS 22.0 (IBM, Armonk, NY, USA).

## 3. Results

### 3.1. General Characteristics of the Participant

**Figure 2:**
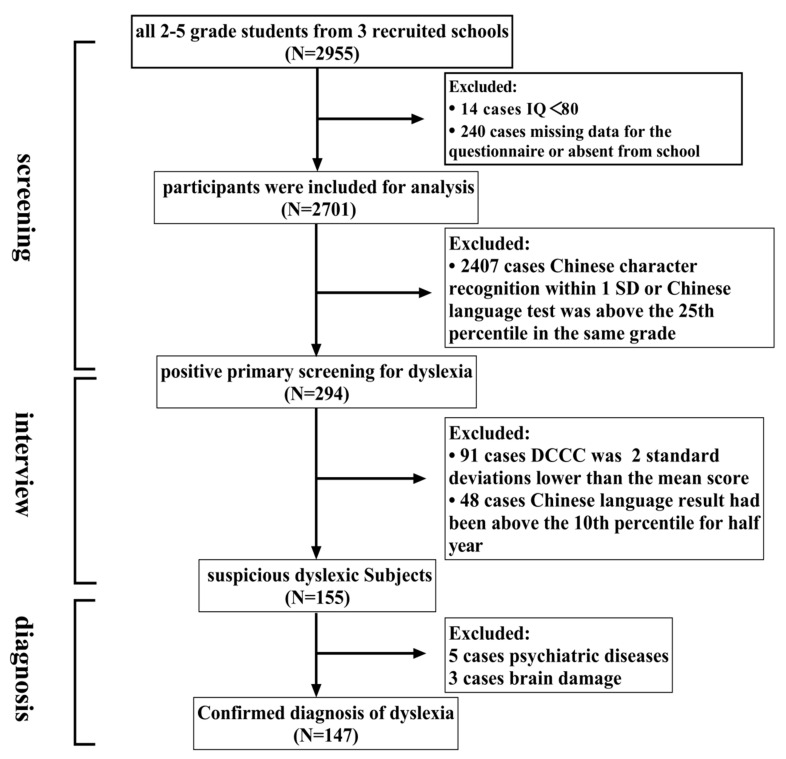
The diagnosis process and results of children with dyslexia in Shantou, China. IQ: Intelligence Quotient; SD: standard deviation; DCCC: Dyslexia Checklist for Chinese Children.

There were 2955 students recruited from grade 2 to grade 5 in the selected schools, based on the cluster sampling. After the exclusion of children with a Raven’s intelligence quotient (IQ) below 80 (*n* = 14), and missing data for the questionnaire or absence from school (*n* = 240), a total of 2701 students who finished all the screening tests were included in the final analysis, with a response rate of 91.4% (Figure 2). The descriptive characteristics of the participants in our study were shown in Table 1. Of the 2701 participants, 1380 (51.1%) were boys and 1321 (48.9%) were girls, aged from 7 to 12 years, with a mean age of 9.07 years.

### 3.2. The Prevalence Rates of Dyslexia in Different Genders and Grades

Based on the three stages of investigation, 147 participants were finally diagnosed as dyslexic, and the prevalence rate of dyslexia was calculated to be 5.4% in Shantou, China. As shown in Table 2, it was found that the prevalence rate of dyslexia was significantly higher in boys than in girls (8.4% vs. 2.3%, *p* < 0.001). The prevalence rates of dyslexia from grade 2 to grade 5 were 6.7%, 5.4%, 4.4%, and 6.1%, respectively, without significant differences of prevalence rates across the four grades (*p* > 0.05).

### 3.3. The Evaluation Results of CRAT

A total of 81 children diagnosed as dyslexic, according to the DSM-5, finished the CRAT scales evaluation, and 103 normally developing children were also recruited as controls to participant in the CRAT evaluation. Table 3 shows the results of the CRAT evaluation between the dyslexia group and the control group. The scores of the five subscales and corresponding items of the CRAT were all significantly lower in children with dyslexia than in the control group (all *p* < 0.001).

### 3.4. Multivariate Logistic Regression Analysis of Chinese Reading Ability Associated with Dyslexia

To estimate the effects of Chinese reading abilities with dyslexia, we performed a multivariate logistic regression analysis. From Table 4, we could see that phonological awareness (odds ratio (OR) = 0.91, 95% CI: 0.09–0.98), rapid automatized naming (OR = 0.19, 95% CI: 0.08–0.46), and orthographic awareness (OR = 0.81, 95% CI: 0.68–0.96) were negatively associated with children’s dyslexia.

## 4. Discussion

Research on dyslexia is growing in China, but public awareness of dyslexia is seriously inadequate. In the process of teaching reading, schools and parents do not have enough cognition of children with dyslexia, and they mistakenly equate dyslexia with poor learning attitudes and intellectual impairment. The United Kingdom and Canada have introduced a series of laws and regulations to guarantee the fair rights and interests of people with dyslexia [24,25], but there are few relevant laws on dyslexia in China. In the United States, 30 states have laws to improve screening for dyslexia [26]. According to what we know, epidemiological investigation of dyslexia was only carried out in the central and southern cities of China. This study used a newly developed assessment tool named CRAT combined with the DSM-5 by child psychiatrists to assess the prevalence of dyslexia in primary school children in Shantou city, China, as well as their reading ability. We screened 2701 children (1380 males and 1321 females) from three primary schools, and found that the prevalence of Chinese dyslexia was 5.4% in Shantou, with a male-to-female ratio of 3.7:1. The children with dyslexia scored lower in all the reading ability tests by CRAT than the normally developing children.

Chinese researchers have suggested that a reasonable assessment of dyslexia must meet the definition of the International Dyslexia Association and reflect Chinese characteristics [13]. However, researchers in mainland China have different screening methods for dyslexia, leading to differences in the prevalence reported. A previous study, which included 6350 students from the second to the sixth grades, suggested that the prevalence of dyslexia was 3.9% in Qianjiang city (middle China) [17]. Recently, another epidemiological survey showed that 4.3% of children in fifth grade were screened as having a reading disability in the city of Nanning (southern China) [20]. Both of the two studies applied the low achievement criteria. In our study, we reported the prevalence rate of 5.4%, which is higher than those of the above two studies. The difference may be caused by the diversity of the study population and diagnostic criteria. Compared with our study, the reported prevalence of dyslexia in Hong Kong was 9.7 % and in Taiwan was 7.5 %, which were higher than those in the above studies [14]. In fact, simplified Chinese characters are used in mainland China, while traditional Chinese characters are used in Hong Kong and Taiwan. Due to traditional Chinese characters having more complicated orthography than the simplified Chinese characters, the difference of two types of Chinese characters might explain the difference in the prevalence of dyslexia.

Most of the research has suggested that the prevalence of dyslexia in alphabetic languages, which was 5% to 17%, was higher than that in non-alphabetic languages [12]. A cross-sectional study in Xinjiang province (Northwest China) may explain the phenomenon. Uygur, a Turkic family of phonetic languages, is similar to alphabetic languages [27]. The researchers found that Uyghur children (7.0%) had a higher proportion of dyslexia than Han children (3.9%) in five Uyghur bilingual primary schools [18]. In addition, studies have shown that Chinese and English dyslexia have different neural bases using structural neuroimaging [28,29]. On the other hand, Siok et al. proposed a new idea—that the biological abnormality of dyslexia is due to cultural differences [30]. In fact, Chinese is a non-alphabetic language, which may lead to different results in dyslexia, compared with an alphabetic language. Our findings basically support the view that the prevalence of dyslexia in China is lower than that reported in English-speaking countries.

Similar to previous findings, we observed that the risk of dyslexia in male children was significantly higher than in female children (3.7:1) [14,17,18,19,31,32]. There is also a different view that the increased prevalence of dyslexia in boys, compared with girls, reflects a bias in subject choice. Shaywitz et al. point out that reports of rising prevalence of dyslexia in boys may be due to the almost inevitably large referral bias in the sample diagnosed in schools. [33]. However, Rutter et al. [34] compared details of gender differences in dyslexia from four separate epidemiological studies, and again emphasized that dyslexia is significantly more common among boys than girls. Katusic et al. conducted a cohort study of 5718 children born between 1976 and 1982. The observations showed that boys were more likely to develop dyslexia than girls, regardless of the identification method [16]. Moreover, recent genetic studies have also found evidence that the CNTNAP2 gene variant is associated with gender differences among dyslexia children in China [15]. Based on the above evidence, we are more inclined to support the conclusion that men are at higher risk for dyslexia than women. Our results showed the prevalence rates of dyslexia within grades were not statistically different. A few studies have demonstrated that the students in lower grades are much more easily affected than those in higher grades [12,17]. However, other studies have had the opposite opinion, that there is no association between grades and the prevalence of dyslexia [19], which is consistent with our results. Therefore, we believe that the incidence of dyslexia is relatively stable in all grades.

Reading is a complex process, involving the integration of many language processing abilities, especially cognitive–linguistic skills. Our study found that dyslexic children had lower levels in the five Chinese cognitive–linguistic skills compared to normal children. As far as we know, this is the first study to explore the relationship between dyslexia and cognitive–linguistic skills in mainland China. Language processing deficits are commonly reported in dyslexia, including deficits of phonological awareness, morpheme awareness, orthographic awareness, and rapid automatic naming [35,36,37], which is in line with our findings. According to our results, compared with the control group, the scores for rhyme, onset, and tone identification in the dyslexic children were significantly lower. It shows that children with dyslexia have a lack of phonological awareness and the ability to transform character symbols into phonetic symbols. Our results also show that children with dyslexia lack the ability to perceive and recognize speech clarity, which may be one of the main reasons for reading and writing difficulties. The results of the word formation awareness subscale indicate that the word formation test took longer, and the scores were significantly lower in the case group, suggesting that the children with dyslexia had the disorder for word formation awareness. From the results of the rapid naming test, we found that the time consumption of the case group was significantly higher than that of the control group, demonstrating that the children with dyslexia had the disorder for naming speed, which may be related to speech processing ability and auditory processing impairment. Orthographic awareness refers to the ability to understand writing habits in a particular language and to distinguish whether writing is correct or not. The scores of non-character recognition and component recognition in the case group were much lower than those in the control group. Apparently, children with dyslexia had abnormal discrimination ability in the structure and writing of Chinese characters, and lacked the regular awareness of characters. Reading comprehension tests included reading time, as well as oral and written questions. In our research, the correct reading numbers by children with dyslexia per minute were significantly lower than the control group. Children with dyslexia also needed to spend much more time on reading, increasing the total reading time and completion time, and had significantly lower scores, demonstrating that they had universal difficulty in reading comprehension, slow speed, and low accuracy of reading.

Multivariate logistic regression analysis has suggested that phonological awareness, rapid automatic naming, and orthographic awareness were significantly correlated with dyslexia. Impairment of cognitive–linguistic skills is closely related to the occurrence of dyslexia, but there has been no conclusive debate on which kind of cognitive–linguistic skill is the core deficit of Chinese dyslexia. It has been well-established that the deficit of phonological awareness is a causal risk factor for dyslexia for alphabetic languages [36,37,38]. However, several findings in Hong Kong have indicated that the deficit of morphological awareness was a relatively strong correlate of dyslexia in Chinese, but phonological awareness was not [39]. In our study, the results suggest that phonological awareness was significantly negatively associated with dyslexia, which was contrary to the previous reports from Hong Kong [6,39]. This difference might be due to the discrepancy in teaching methods between Hong Kong and mainland China. In mainland China, pinyin teaching was the primary Chinese linguistic education mode, and children from kindergarten to grade 2 of primary school paid more attention to pinyin education; therefore, phonological awareness was closely related to children’s early reading development. Besides, evidence from an eight-year, longitudinal study has suggested that phonological awareness provides the basis for the development of morphological awareness in Chinese, which is a better indicator for predicting children’s future reading development [40]. In addition, studies have revealed that rapid automatized naming deficits play an important role in predicting the incidence of dyslexia [35,37], which is consistent with our results. Rapid automatic naming was strongly associated with early childhood reading skills, and it might be related to speech processing ability and auditory processing impairment. Finally, orthographic awareness was also significantly associated with dyslexia in multivariate logistic regression analysis, which is similar to other studies [41]. Apparently, children with dyslexia had abnormal discrimination ability in the structure and writing of Chinese characters, and lacked the regular awareness of characters. Previous studies have suggested that the core deficit of dyslexia is simply phonological awareness or morpheme awareness [39,42,43]. In fact, the researchers tend to believe that the core deficit of the cognitive–linguistic skill in dyslexia is not unilateral, and it might be dual or multiple deficits. Our results support the multiple-deficits hypothesis, and suggest that the deficits of phonological awareness, rapid automatic naming, and orthographic awareness are the main deficits in Chinese dyslexia. In the univariate analysis, the scores of morpheme awareness and reading comprehension for dyslexic children were significantly lower than those of the control group, which indicates that the dyslexic children are indeed deficient in morpheme awareness and reading comprehension. In fact, children with dyslexia may have multiple cognitive deficits simultaneously, including phonological awareness, morpheme awareness, rapid nomenclature, and orthographic awareness. However, in multivariate logistics regression analysis, our results showed that there were no significant differences in morpheme awareness and reading comprehension. Therefore, we believed that morpheme awareness and reading comprehension were potential influencing factors rather than the core defects of dyslexia.

### Strength and Limitations

Reading ability level tests are important, aiding in confirmation for the diagnosis of dyslexia. In this study, not only did we use the screening scales (CRT, CVTAS, DCCC, and DSM-5) to screen and confirm dyslexia, but also adopted newly developed CRAT for the reading ability level test. Our results may provide more information and b closer to the true prevalence rate of dyslexia in Chinese-speaking primary school children in China. However, there are still some limitations in this study. The study was carried out in one city in China, and it needs to be expanded to more cities to reflect the prevalence of Chinese dyslexia. The CRAT is only applicable to students above grade 3; the lower grade students are not competent to complete it perfectly, therefore their data were not included in the final CRAT analysis, which may lead to some selection bias. In addition, we did not examine the effect of academic performance on dyslexia, and lack control for sociodemographic variables in data analysis.

## 5. Conclusions

This study investigated the prevalence of DD in primary school children in southeast China, as wll as their Chinese reading ability. The rough prevalence rate of children with dyslexia was reported as 5.4% in our samples, with much lower Chinese reading ability than normally developing children. In summary, it is necessary to investigate the prevalence rate of Chinese dyslexia, with a more scientific and appropriate method for meeting the definition of dyslexia. The prevalence of Chinese dyslexia is lower than that in English-speaking countries, but the number of children in China is huge, which should increase public awareness of dyslexia.

## Figures and Tables

**Table 1:** Descriptive statistics of the participants (*N* = 2701).

Variables	*N*	%
Non-dyslexia	2554	94.6
Dyslexia	147	5.4
Gender		
Boy	1380	51.1
Girl	1321	48.9
Age(years), mean ± SD	9.07 ± 1.09	
Grade		
Grade 2	372	13.8
Grade 3	746	27.6
Grade 4	878	32.5
Grade 5	705	26.1

**Table 2:** The prevalence rates of dyslexia in different gender and grades.

Characterictics	Dyslexia (*N* = 147)	No Dyslexia (*N* = 2554)	Prevalence Rate (%)	*χ* ^2^	*p*
Gender				48.149	<0.001
Boy	116	1264	8.4		
Girl	31	1290	2.3		
Grade				3.489	0.322
Grade 2	25	347	6.7		
Grade 3	40	706	5.4		
Grade 4	39	839	4.4		
Grade 5	43	662	6.1		

**Table 3:** The result of the Chinese Reading Ability Test (CRAT) in the dyslexia group and the control group.

Variable	Dyslexic (*N* = 81)	Normal (*N* = 103)	*t*	*p*
Phonological awareness	21.00 ± 7.19	28.72 ± 5.35	8.067	<0.001
Tone scores	7.49 ± 3.56	10.33 ± 2.80	−6.048	<0.001
Onset scores	7.26 ± 2.73	9.54 ± 1.70	−6.944	<0.001
Rime scores	6.25 ± 2.83	8.84 ± 2.24	−6.951	<0.001
Morphological awareness	8.53 ± 1.79	9.84 ± 0.54	6.333	<0.001
Chinese word formation time (s)	185.53 ± 65.38	148.16 ± 33.98	5.006	<0.001
Chinese word formation scores	8.53 ± 1.79	9.83 ± 0.54	−7.001	<0.001
Rapid automatized naming	2.20 ± 0.61	2.85 ± 0.49	7.888	<0.001
Time	19.78 ± 7.11	14.59 ± 2.59	6.851	<0.001
Number of wrong words in reading	0.42 ± 0.89	0.23 ± 0.55	1.758	<0.001
Orthographic skills	24.59 ± 3.25	27.42 ± 3.25	6.810	<0.001
Non-character recognition scores	15.54 ± 2.17	16.35 ± 1.61	−6.479	<0.001
Radical position time	41.86 ± 20.11	33.81 ± 19.18	2.768	<0.001
Radical position score	10.05 ± 1.91	11.07 ± 1.12	−4.513	<0.006
Reading comprehension	8.85 ± 2.96	10.74 ± 2.02	−5.141	<0.001
Number of words in 1 minute of reading	160.13 ± 48.94	220.03 ± 41.46	−8.957	<0.001
Time for reading an article	125.70 ± 57.57	84.65 ± 27.72	6.306	<0.001
Total score of reading comprehension	8.85 ± 2.96	10.74 ± 2.02	−5.141	<0.001

**Table 4:** Binary logistic regression analysis of five Chinese cognitive–linguistic skills for dyslexia.

Variables	*β*	SE	Wald	OR	95% CI	*p*
Phonological awareness	−0.091	0.036	6.163	0.91	(0.09–0.98)	0.013
Morphological awareness	−0.492	0.289	2.893	0.35	(0.35–1.08)	0.089
Rapid automatized naming	−1.653	0.449	13.536	0.19	(0.08–0.46)	<0.001
Orthographic awareness	−0.210	0.088	5.671	0.81	(0.68–0.96)	0.017
Reading comprehension	−0.069	0.097	0.503	0.93	(0.77–1.13)	0.478

*β*: regression coefficient; SE: standard error; OR: odds ratio; CI: confidence interval.
